# The Role of Psychological Distress on Health‐Related Quality of Life, Fatigue, and Pain in Adults With Pulmonary Hypertension

**DOI:** 10.1002/pul2.70101

**Published:** 2025-06-17

**Authors:** Gregg H. Rawlings, Abbie Stark, Iain Armstrong, Vlad Costin, Andrew R. Thompson

**Affiliations:** ^1^ Clinical and Applied Psychology Unit University of Sheffield Sheffield UK; ^2^ Cardiff and Vale University Health Board and Cardiff University Glamorgan UK; ^3^ Sheffield Pulmonary Vascular Disease Unit, Royal Hallamshire Hospital, Sheffield Teaching Hospitals NHS Foundation Trust Sheffield UK; ^4^ School of Psychology University of Sussex Brighton UK

**Keywords:** anxiety, depression, mental health, psychology, pulmonary arterial hypertension

## Abstract

While anxiety and depression are commonly reported in pulmonary hypertension (PH), limited evidence exists on how these conditions interact with the pathophysiological symptoms of PH. Fatigue and, to a lesser degree, pain are key symptoms of PH; however, they have rarely been examined as separate experiences associated with PH. Using a cross‐sectional research design, 68 adults with PH recruited from global Pulmonary Hypertensions Associations completed a series of self‐report measures assessing fatigue, pain self‐efficacy, anxiety, depression, and health‐related quality of life (HRQoL). Aiming to understand the nuances of PH symptomatology, we first looked at responses on individual items from fatigue and pain measures, respectively. Then, to examine relationships between self‐reported symptoms, we tested potential pathways from fatigue and pain to HRQoL through depression and anxiety. All symptoms were correlated, suggesting individuals with greater anxiety and depression also experienced more fatigue, and lower pain self‐efficacy and HRQoL. Parallel mediation analyses showed that fatigue and pain had a direct effect on HRQoL, as well as an indirect effect via anxiety and depression. Explorative serial mediation models suggested the indirect path from fatigue to HRQoL was significant when depression was ordered first followed by anxiety; whereas for pain self‐efficacy, the path was significant when anxiety was followed by depression. Results add to the evidence demonstrating the high co‐occurrence of mental health difficulties in PH and the important role they play in pathophysiological symptomatology. Analyses support providing holistic care for this clinical group to help identify various therapeutic targets suggestive of predicting HRQoL.

## 1 Introduction

Pulmonary hypertension (PH) is a group of serious and debilitating conditions characterised by elevated pulmonary artery pressure at rest. There are five different groups of PH: (i) pulmonary arterial hypertension (PAH), which is considered a rare condition; (ii) PH associated with left heart disease and (iii) PH associated with lung diseases and/or hypoxia, which are more common; and (iv) PH associated with pulmonary artery obstructions and (v) PH with unclear and/or multifactorial mechanisms, which are also seen as rare [1, 2]. PH is associated with high rates of early mortality [3] and morbidity, which includes physiological [4], psychological [5, 6], cognitive [7] and emotional difficulties [8, 9, 10, 11]. Indeed, it is generally accepted that people with PH are at a higher risk of experiencing anxiety and depression than the general population, with pooled rates of 37% and 28% being reported respectively [12]. These difficulties in turn have been associated with reduced health‐related quality of life (HRQoL) [8, 9, 13, 14, 15]. While much of the research has predominantly involved adults with PAH, rates appear to be generally high across different types of PH [12].

A relatively considerable body of evidence exists that has utilised qualitative methods to better understand the phenomenology of PH. Much of this data was synthesised in a systematic review in 2020, which included the personal accounts of over 1900 adults with PH across Europe, North and South America, and Asia. Amongst participants' descriptions, fatigue and, to a lesser extent, pain appeared common and were reported as having a negative impact on daily life [16]. The high prevalence of fatigue and pain in PH is further supported by quantitative methods of inquiry. For example, patient‐reported outcome measures in PH specifically include questions related to either or both of these symptoms [17]. This includes the PAH Symptoms Inference Scale (PAHSIS), which when administered to 191 individuals with PAH, fatigue was reported by 90% (second only to shortness of breath on exertion, 92%) and chest pain by 56%. Moreover, while fatigue was only measured using one‐item, it was found to be a significant predictor of HRQoL as measured using the Medical Outcomes Study Short Form‐36 (SF‐36) [18]. Similar rates were found in another study involving 67 patients with PAH who were asked whether they have experienced a list of 47 common PH‐related difficulties. 87.9% experienced fatigue while 40.6% reported pain [19]. Fatigue and pain appear to be a common feature of PH regardless of how far the disease has progressed as they are some of the most frequently reported symptoms by patients at symptom onset for PH and the reason why they first seek medical help [20].

Fatigue is not specific to PH as it has been shown to be a common symptom in those living with a range of chronic illnesses [21]. Fatigue has been conceptualised as a subjective experience, which can be characterised by tiredness, exhaustion, lack of energy, weakness, and reduced concentration. Some researchers have dichotomised fatigue‐related experiences into physical or mental symptoms. Fatigue‐related symptoms can be debilitating in their own right but can also have a negative impact on different aspects of daily life [22]. Specific unidimensional and multidimensional scales have been developed to measure fatigue [23]; however, they have seldom been used in PH populations despite its prevalence [24, 25].

Tartavoulle and colleagues asked 120 patients with PH to complete the Multidimensional Fatigue Inventory (MFI‐20) [24]. While overall symptoms of fatigue were high, with the majority rating their fatigue as *severe* or *very severe* (60%), the severity of some fatigue‐related symptoms were greater than others: 55.8% rated their physical symptoms of fatigue as *severe* or *very severe*, 41.7% reported severely reduced activity, 32.5% severely reduced motivation and 27.5% severe mental fatigue. Severity of fatigue was not associated with age, sex, ethnicity, or type of PH; however, significant differences were observed between groups categorised by World Health Organisation (WHO) functional class, as all fatigue‐related symptoms were more severe in those with class III/IV compared to those with class I/II.

Pain is also a common experience reported by people living with a long‐term health condition [26]. Like fatigue, pain has been viewed as a subjective experience that can be multifaceted. Specific tools to measure proposed dimensions of pain have been developed, including the pain self‐efficacy questionnaire. This measure is based on the concept that an individual's belief in their pain is central to how it is experienced and reported. How confident a patient is in their ability to cope with pain and its impact has been shown to be a strong predictor of treatment outcome [27]. We are unaware of any published study that has asked patients with PH to complete a self‐report measure that is specific to any aspect of pain, including pain self‐efficacy.

The link between fatigue, pain, psychological distress, and HRQoL has been examined in depth in other chronic cardiovascular conditions. For example, in individuals with chronic obstructive pulmonary disease (COPD), for whom depression and anxiety are also common, depression and anxiety influenced symptoms of fatigue. Fatigue was also observed as independently impacting health status [28]. A systematic review on COPD identified pain as being correlated with HRQoL, fatigue, anxiety, depression insomnia, breathlessness, and nutritional status [29]. In 167 patients with heart failure, pain was the strongest predictor of the physical component of a HRQoL measure; in comparison, pain was not a significant predictor of the mental component [30].

Although the high prevalence of fatigue and pain in PH has been well documented, their impact, how people cope with these symptoms and to what degree they interlink with other symptoms remains relatively unexplored. The first aim of the current study was to descriptively report participant's responses to multi‐item measures of fatigue and pain. Indeed, most research to date has examined these symptoms as part of a larger measure, whereby experiences are assessed via one item and/or participant's responses are grouped with other questions to create an overall score. This has limited our ability to develop a nuanced understanding of these symptoms in this clinical group. Our second aim was to examine the interrelations between fatigue, pain self‐efficacy, HRQoL and measures of psychological distress, namely anxiety and depression. We predicted that both fatigue and pain self‐efficacy would correlate with anxiety, depression and HRQoL. Following this, we specified two parallel mediation models to test whether depression and anxiety would partially explain the effects of fatigue (Model 1) or pain (Model 2) on HRQoL. It was hypothesised that anxiety and depression would partially mediate the relationship; however, pain self‐efficacy and fatigue would still have a direct impact on HRQoL after controlling for their indirect effects through anxiety and depression.

## 2 Methods

### 2.1 Design

A cross‐sectional research design was utilised. Data was collected as part of a randomised controlled trial design testing the effectiveness of a cognitive behavioural self‐help intervention for depression in adults with PH [31]. This study had obtained ethical approval from the University Psychology Ethics Committee at Cardiff University (EC.22.12.13.6673R2A). The data analysed here was collected at baseline, before participants being randomised to receive the intervention or to a waitlist group. Participants provided consent for their data to be used for future research. Ethical approval for the current study was also obtained from the School of Psychology at the University of Sheffield (064631).

### 2.2 Participants and Procedure

A volunteering sampling method was used as participants responded to an invitation promoted by Pulmonary Hypertension Associations (PHA) around the world, including PHA UK. After reading a participant information sheet and completing a consent form, participants were asked to complete a series of outcome measures (see below). Participants must have self‐reported a difficulty with depression; participants were not eligible if they were experiencing thoughts of suicide. Participants with any type of PH or any WHO functional class were eligible to take part. All data were collected via the online platform, Qualtrics [32].

### 2.3 Measures

Participants were asked to complete a demographic questionnaire capturing their age, gender, ethnicity, years of education, and employment status. Participants were also asked to self‐report the number of years since their diagnosis of PH, PH type, and functional class – participants were given the option to select “not sure” if they did not know this information.

Fatigue was assessed using the 9‐item Fatigue Severity Scale (FSS) [33]. Participants were asked to rate their symptoms in the previous week using a seven‐point Likert scale. Summed scores ranged from 9 to 63 with higher scores suggesting greater severity of fatigue symptoms [34]. Cronbach's alpha was 0.94 (Cronbach's alpha ≥ 0.9 = excellent, ≥ 0.8 = good, ≥ 0.7 = acceptable, ≥ 0.6 = poor, ≤ .5 = unacceptable [35].

Pain was measured using the Pain Self‐Efficacy Questionnaire (PSEQ) [36]. This is a ten‐item measure that aims to assess how confident people feel about undertaking routine activities while in pain. Scores range from 0 to 60. A higher sore suggests greater confidence and self‐efficacy in dealing with their pain. A score of 40 or more predicts a good response to self‐management and return to work [27]. The PSEQ is suggested to be applicable to any individual with persistent pain. Cronbach's alpha was 0.94.

Depression was measured using the Patient Health Questionnaire‐8 (PHQ‐8) [37]. Participants were asked to rate how often they have experienced eight depression‐related symptoms over the last 2 weeks. A higher score suggests more severe depression. Scores can range from 0 to 24 and a cut off of 10 is proposed. The PHQ‐9 (which is comprised of the same eight items plus one additional question related to thoughts of suicide or self‐harm) has been administered to people with PH previously [38]. Cronbach's alpha was 0.86.

Anxiety was investigated using the Generalised Anxiety Disorder‐7 Questionnaire (GAD‐7) [39]. This is a seven‐item measure asking respondents to rate a series of anxiety‐related symptoms in the context of the last 2 weeks. Scores range from 0 to 21 with a higher score suggesting more severe anxiety. A cut off of 8 is proposed. The GAD‐7 has been used previously in patients with PH [38]. Cronbach's alpha was 0.93.

Finally, HRQoL was measured using the emPHasis‐10 [40]. This is a PH specific HRQoL measure that is heavily used in research and clinical settings. The emPHasis‐10 consists of ten items related to challenges that people with the condition have identified as negatively impacting on their quality of life. Scores can range from 0 to 50 with a higher score suggesting lower HRQoL. Cronbach's alpha was 0.93. While the emPHasis‐10 is a unidimensional measure, it has recently been proposed that three factors can be calculated based on participant's scores, namely fatigue (Cronbach's alpha = 0.79), breathlessness (Cronbach's alpha = 0.75) and independence (Cronbach's alpha = 0.81) [41].

### 2.4 Data Analysis

Data clean up and item‐level descriptive statistics were obtained using IBM SPSS Statistics 28 [42]. All other analyses were performed in R v4.4.1 [43] and mediation analyses were completed using the lavaan package (v0.6.17) [44]. Mediation analyses were performed using maximum likelihood estimation. We tested normality using the MVN R package (v5.9) [45]. Mardia's [46] test of multivariate normality, which suggested we can assume multivariate normality for the mediation models involving pain self‐efficacy, but not for the mediation models involving fatigue (Mardia skewness *p* = 0.009). In addition, a series of Shapiro‐Wilk tests suggested we cannot assume univariate normality for fatigue (*p* < 0.001), anxiety (*p* = 0.004) and HRQoL (*p* = 0.041); these results were confirmed by inspecting the histograms and Q‐Q plots which showed a slight negative skew for fatigue and HRQoL and a positive skew for anxiety. To address this, mediation analyses were specified using bootstrapped standard errors (1000 samples), which are robust to violations of normality [47].

As the mediation models involved multiple simultaneous predictors, we tested the assumption of multicollinearity by inspecting correlations between predictors in Model 1 (fatigue, anxiety, and depression) and Model 2 (pain self‐efficacy, anxiety, and depression), respectively. In both cases, predictors were moderately correlated but captured distinct constructs (rs ≤ 0.55; see Table 4).

## 3 Results

### 3.1 Participants

In total, 68 individuals with PH completed the measures. Most were female, self‐reported their ethnicity as White and retired. The mean age of participants was 52.5 years with a range of 53 years (28–81 years of age). Years in education was high with the average participant remaining in education until after secondary education, that is, college or university. Although the mean years of living with PH was relatively high, the variance was large with a range of 49 years. Most individuals reported being diagnosed with idiopathic PH; the second largest group reported not knowing their diagnosis or did not share this information. Similarly, the largest group of individuals did not report their WHO functional class; of those who did record their class, class II and III were the most prevalent.

In terms of patient reported outcome measures, the overwhelming majority of participants scored above the clinical off for fatigue (86.8%) suggesting they should seek additional support. Indeed, the average was well above the cut off value. Responses to the PSEQ suggested only 22.1% had a good response to self‐management; the average was below the cut off. Although this was a community sample, 73.5% and 58.8% scored above the clinical cut off for depression and anxiety, respectively. Scores on the HRQoL were slightly above the midpoint of possible scores (0–50); scores for fatigue, breathlessness and independence as measured by the emPHasis‐10 had a similar variance (Table 1).

**Table 1:** Summary of participants (*n* = 68).

	Mean (standard deviation)	*N* (%)
Demographic		
Gender		
Female		58 (85.3%)
Male		10 (14.7%)
Age (years)	52.49 (12.86)	
Ethnicity		
White		54 (79.4%)
Asian		5 (7.4%)
Black		1 (1.5%)
Mixed or multiple ethnic groups		3 (4.4%)
Not reported		5 (7.4%)
Years in education	15.46 (5.1)	
Employment status		
Full time		21 (30.9%)
Part time		8 (11.8%)
Not employed		16 (23.5%)
Retired		23 (33.8%)
Clinical information		
Years with PH	9.2 (10.7)	
Type of PH		
Idiopathic PH		22 (32.4%)
Connective tissue disease		6 (8.8%)
Chronic thromboembolic PH		15 (22.1%)
Familial PH		1 (1.5%)
Congenital PH		7 (10.3%)
Other		1 (1.5%)
Not reported/don't know		16 (23.5%)
WHO functional class		
I		6 (8.8%)
II		14 (20.6%)
III		16 (23.5%)
IV		4 (5.9%)
Not reported/don't know		28 (41.2%)
Patient reported outcome measures		
Fatigue	51.74 (11.33)	
Above cut off (> 36)		59 (86.8%)
Pain self‐efficacy questionnaire	28.71 (13.27)	
Below cut off (> 40)		15 (77.9%)
PHQ‐8	13.16 (5.33)	
Minimal depression (0–4)		4
Mild (5–9)		14
Moderate (10–14)		23
Moderately severe (15–19)		19
Severe (20–24)		8
Above cut off (> 10)		50 (73.5%)
GAD‐7	10.26 (5.9)	
Minimal anxiety (0–4)		11 (16.2%)
Mild (5–9)		24 (35.3%)
Moderate (10–14)		17 (25%)
Severe (15–21)		16 (23.5%)
Cut off (> 8)		40 (58.8%)
emPHasis‐10	31.15 (9.4)	
Fatigue	10.75 (3.33)	
Independence	10.87 (4.32)	
Breathlessness	9.53 (3.25)	

Abbreviations: GAD, Generalised Anxiety Disorder; HRQoL, Health‐related quality of life; N, Number; PHQ, Patient Health Questionnaire; PH, Pulmonary hypertension; WHO, World Health Organisation.

### 3.2 Descriptive Profile of Fatigue and Pain Self‐Efficacy in PH

Most participants agreed (scored five or greater) with all of the nine‐fatigue related items suggesting symptoms were frequently experienced. The most common symptom participants experienced was that fatigue would impact on their level of motivation. The symptom with the lowest agreement, although still endorsed by 69.1%, was whether exercise was associated with fatigue. Approximately 80% agreed their fatigue interfered with their physical functioning, duties, responsibilities, and in their personal and professional life. 80% reported fatigue as among their three most disabling symptoms (Table 2).

**Table 2:** Distribution of scores on the Fatigue Severity Scale. Values reflect n unless otherwise stated.

	Disagree (1–3)	Neutral (4)	Agree (5–7)
My motivation is lower when I am fatigued.	1 (1.5%)	1 (1.5%)	66 (97%)
Exercise brings on my fatigue.	14 (20.6%)	7 (10.3%)	47 (69.1%)
I am easily fatigued.	5 (7.4%)	12 (17.6%)	51 (75%)
Fatigue interferes with my physical functioning.	5 (7.4%)	7 (10.3%)	56 (82.3%)
Fatigue causes frequent problems for me.	9 (13.2%)	5 (7.4%)	54 (79.4%)
My fatigue prevents sustained physical functioning.	9 (13.2%)	7 (10.3%)	52 (76.5%)
Fatigue interferes with carrying out certain duties and responsibilities.	10 (14.7%)	4 (5.9%)	54 (79.4%)
Fatigue is among my three most disabling symptoms.	7 (10.3%)	6 (8.8%)	55 (80.9%)
Fatigue interferes with my work, family, or social life.	7 (10.3%)	3 (4.4%)	58 (85.3%)

Compared to fatigue, experiences related to pain were more heterogeneous and pain was generally less common. The majority of participants (> 50%) felt confident they could enjoy things whether they experienced pain and could cope. Most (85.2%) either felt confident or did not feel confident to manage their pain without medication, suggesting a considerable split in patient's perceptions. The largest proportion of people did not feel confident that they could be social with others as often as they used to, do many of the things they enjoy, accomplish most of their goals, live a normal lifestyle or gradually become more active (Table 3).

**Table 3:** Distribution of scores on the Pain Self‐Efficacy Measure. Values reflect n unless otherwise stated.

	Not confident (0–2)	Neutral (3)	Confident (4–6)
I can enjoy things, despite the pain.	12 (17.6%)	20 (29.4%)	36 (52.9%)
I can do most of the household chores (e.g., tidying‐up, washing dishes, etc.), despite the pain.	25 (36.8%)	17 (26.4%)	25 (36.8%)
I can socialise with my friends or family members as often as I used to do, despite the pain.	33 (48.5%)	16 (23.5%)	19 (28%)
I can cope with my pain in most situations.	17 (25.3%)	14 (21%)	36 (53.7%)
I can do some form of work, despite the pain. (‘work’ includes housework, paid and unpaid work).	20 (29.4%)	20 (29.4%)	28 (41.2%)
I can still do many of the things I enjoy doing, such as hobbies or leisure activity, despite pain.	33 (49.3%)	15 (22.3%)	19 (28.4%)
I can cope with my pain without medication.	30 (44.1%)	10 (14.7%)	28 (41.2%)
I can still accomplish most of my goals in life, despite the pain.	28 (41.2%)	16 (23.5%)	24 (35.3%)
I can live a normal lifestyle, despite the pain.	33 (48.5%)	14 (20.6%)	21 (30.9%)
I can gradually become more active, despite the pain.	32 (47.1%)	15 (22.1%)	21 (30.8%)

### 3.3 Interrelations Between Fatigue, Pain Self‐Efficacy, HRQoL, Depression, and Anxiety

Fatigue and pain self‐efficacy were significantly correlated with all self‐reported measures of functioning, namely anxiety, depression, and HRQoL. In other words, those who experienced lower HRQoL, and greater symptoms of anxiety and depression, were more likely to experience fatigue and feel less confident about coping with their pain. Age, years of education, or years with PH were not correlated with any of the experiences measured (Table 4).

**Table 4:** Correlation matrix of measures of functioning, demographics, and years living with PH. Values in square brackets indicate the 95% confidence interval.

Variable	1	2	3	4	5	6	7
1. Fatigue							
2. Pain self‐efficacy	−0.64***						
	[−0.76, −0.48]						
3. HRQoL	.70***	−0.60***					
	[.56, 0.81]	[−0.74, −0.43]					
4. Depression	.55***	−0.42***	.66***				
	[.36, 0.70]	[−0.60, −0.20]	[.50, 0.78]				
5. Anxiety	.32**	−0.35**	.57***	.54***			
	[.08, 0.52]	[−0.54, −0.12]	[.39, 0.71]	[.35, 0.69]			
6. Age	.12	−0.04	.08	.22	−0.21		
	[−0.12, 0.35]	[−0.27, 0.20]	[−0.16, 0.31]	[−0.02, 0.44]	[−0.42, 0.03]		
7. Years in education	.05	.06	−0.05	.07	−0.01	.02	
	[−0.22, 0.31]	[−0.20, 0.32]	[−0.31, 0.21]	[−0.19, 0.33]	[−0.27, 0.25]	[−0.24, 0.29]	
8. Years with PH	−0.01	.04	.04	−0.01	.14	−0.14	.13
	[−0.24, 0.23]	[−0.20, 0.27]	[−0.20, 0.28]	[−0.24, 0.23]	[−0.11, 0.36]	[−0.37, 0.10]	[−0.13, 0.38]

Abbreviations: HRQoL, Health‐Related Quality of Life; PH, Pulmonary Hypertension.

*p < 0.05

** *p* < 0.01

*** *p* < 0.001.

**Figure 1:**
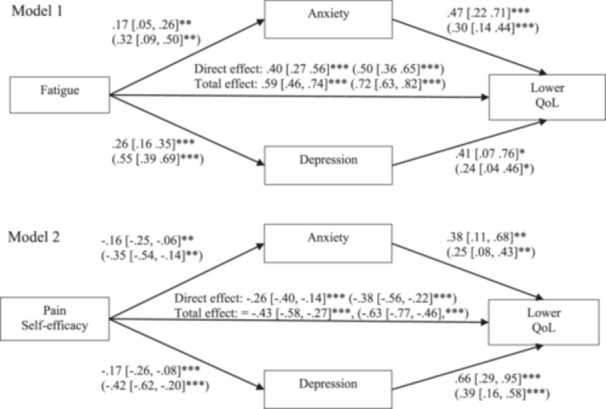
Parallel mediation models showing unstandardised estimates with bootstrapped 95% confidence intervals (and standardised estimates in brackets) for fatigue (Model 1) or pain‐self efficacy (Model 2) on lower HRQoL (health‐related quality of life) through anxiety or depression. *p < 0.05, **p < 0.01, ***p < 0.001.

Parallel mediation analyses were performed to investigate the mediating roles of depression and anxiety in the relationship between fatigue (Model 1) or pain self‐efficacy (Model 2) and HRQoL as shown in Figure 1. As predicted, fatigue significantly predicted HRQoL with indirect effects through anxiety (*b* = 0.08, 95% CI [.01, 0.16], β = 0.10, *p* = 0.028) and through depression (*b* = 0.11, 95% CI [.02, 0.20], β = 0.13, *p* = 0.030), as well as a direct effect (*b* = 0.40, 95% CI [.27, 0.56], β = 0.50, *p* < 0.001). Analyses showed a similar pattern for pain self‐efficacy with significant indirect effects on HRQoL through anxiety (b = −0.06, 95% CI [−0.12, −0.01], β = −0.09, *p* = 0.036) and through depression (b = −0.11, 95% CI [−0.20, −0.04], β = −0.16, *p* = 0.007) and a significant direct effect (b = −0.26, 95% CI [−0.40, −0.14], β = −0.38, *p* < 0.001).

Taken together, the results suggest anxiety and depression partially mediated the relationship between fatigue and HRQoL, and pain self‐efficacy and HRQoL. This indicates that adults with higher levels of fatigue and lower pain self‐efficacy were more likely to have lower HRQoL, which in part, is through having greater symptoms of anxiety and depression.

An additional four serial mediation models were specified to explore the causal chain linking of mediators (anxiety and depression) on HRQoL and fatigue and pain self‐efficacy. As we had no firm hypotheses regarding the causal order of mediators (e.g., fatigue or pain self‐efficacy could increase anxiety, which in turn could increase depression and reduce HRQoL or fatigue or pain self‐efficacy could increase depression, which impact anxiety and lowers HRQoL), both models (anxiety ‐> depression and depression ‐> anxiety) were ran for each predictor variable (fatigue or pain self‐efficacy).

The indirect path of fatigue on HRQoL was significant when depression was ordered first followed by anxiety; the indirect path was not significant when anxiety was the first mediator. The opposite was found for pain self‐efficacy in which, the indirect path was significant when anxiety was first added, but not depression. This means that increased fatigue may result in depressive symptoms contributing to anxiety, which was associated with lower HRQoL, whereas lower pain self‐efficacy was related to greater anxiety, which was related to depression and in turn, lower HRQoL (Table 5).

**Table 5:** Direct and indirect effects of serial mediation models of fatigue or pain‐self‐efficacy on HRQoL with anxiety and depression as mediators.

Model	*b* [95% CI]	*β* [95% CI]	*p*
**Model 1: Fatigue predicting HRQoL**			
Total effect of fatigue on lower quality of life	.59 [.46, 0.74]	.71 [.61, 0.81]	< 0.001
Direct effect of fatigue on lower quality of life	.40 [.27, 0.56]	.48 [.35, 0.65]	< 0.001
**Model 1 A: Anxiety ‐> Depression**			
Indirect effect: Fatigue ‐> Anxiety ‐> Depression ‐> Lower HRQoL	.03 [.00, 0.07]	.03 [.00, 0.08]	.126
Indirect effect: Anxiety ‐> Depression ‐> Lower HRQoL	.15 [.03, 0.34]	.10 [.02, 0.21]	.052
Indirect effect: Fatigue ‐> Anxiety ‐> Lower HRQoL	.08 [.01, 0.16]	.09 [.02, 0.17]	.028
Indirect effect: Fatigue ‐> Depression ‐> Lower HRQoL	.08 [.01, 0.16]	.10 [.02, 0.20]	.033
**Model 1B: Depression ‐> Anxiety**			
Indirect effect: Fatigue ‐> Depression ‐> Anxiety ‐> Lower HRQoL	.07 [.03, 0.14]	.09 [.03, 0.15]	.012
Indirect effect: Depression ‐> Anxiety ‐> Lower HRQoL	.27 [.11, 0.48]	.16 [.06, 0.26]	.003
Indirect effect: Fatigue ‐> Anxiety ‐> Lower HRQoL	.01 [−0.05, 0.07]	.01 [−0.06, 0.08]	.835
Indirect effect: Fatigue ‐> Depression ‐> Lower HRQoL	.11 [.02,0.20]	.13 [.02,0.25]	.030
**Model 2: Pain self‐efficacy predicting HRQoL**			
Total effect of pain self‐efficacy on lower quality of life	−0.43 [−0.58, −0.27]	−0.61 [−0.76, −0.44]	< 0.001
Direct effect of pain self‐efficacy on lower quality of life	−0.26 [−0.40, −0.14]	−0.36 [−0.54, −0.21]	< 0.001
**Model 2 A: Anxiety ‐> Depression**			
Indirect effect: Pain self‐efficacy ‐> Anxiety ‐> Depression ‐> Lower HRQoL	−0.04 [−0.09, −0.01]	−0.06 [−0.12, −0.01]	.037
Indirect effect: Anxiety ‐> Depression ‐> Lower HRQoL	.27 [.01, 0.46]	.17 [.06, 0.30]	.003
Indirect effect: Pain self‐efficacy ‐> Anxiety ‐> Lower HRQoL	−0.06 [−0.12, −0.01]	−0.08 [−0.17, −0.02]	.036
Indirect effect: Pain self‐efficacy ‐> Depression ‐> Lower HRQoL	−0.07 [−0.14, −0.01]	−0.10 [−0.20, −0.01]	.036
**Model 2B: Depression ‐> Anxiety**			
Indirect effect: Pain self‐efficacy ‐> Depression ‐> Anxiety ‐> Lower HRQoL	−0.03 [−0.09, −0.01]	−0.05 [−0.12, −0.01]	.104
Indirect effect: Depression ‐> Anxiety ‐> Lower HRQoL	.20 [.05, 0.43]	.12 [.03, 0.23]	.036
Indirect effect: Pain self‐efficacy ‐> Anxiety ‐> Lower HRQoL	−0.03 [−0.08, 0.02]	−0.04 [−0.11, 0.03]	.276
Indirect effect: Pain self‐efficacy ‐> Depression ‐> Lower HRQoL	−0.11 [−0.20, −0.04]	−0.20 [−0.38, −0.07]	.007

*Note: b,*unstandardised estimates; *β,*standardized estimates; [95% CI],associated bootstrapped 95% confidence intervals; *p,*significance value for the unstandardised path coefficient.

## 4 Discussion

While fatigue and pain have been identified as common features of PH, little is known about how patients are impacted by these symptoms and how they interact with other comorbidities often seen in PH. Our findings are consistent with the wider literature demonstrating the concurrence of fatigue and pain in PH. Although the current sample was recruited from the community and therefore could be assumed to have better controlled symptoms than a sample recruited from a clinical setting, 86.8% reported clinical levels of fatigue and only 22.1% had high (as indicated as above the cut off) self‐efficacy to manage their pain. Our sample also reported high levels of depression (73.5%) and anxiety (58.8%). Greater risk of mood difficulties in PH has been summarised elsewhere [12]; however, these values are above the pooled average for depression and anxiety (28% [95% CI: 20.5–36.8] and 37% [95% CI: 28.7–46.4%] respectively). This might be explained by the fact that our sample were recruited for a psychological intervention targeting depression in PH specifically, and so scores are likely to be elevated at baseline. We found no evidence to suggest fatigue, depression, anxiety, HRQoL or pain self‐efficacy were associated with age, years living with PH, or years of education. While we are aware of research that has reported differences in prevalence of mental health difficulties in PH depending on age [6], generally demographics do not seem to be a reliable predictor of mood disorders in this clinical group [12, 48]. Our findings support a need to screen all patients for these difficulties, regardless of demographics.

Analyses shown lower pain self‐efficacity and greater symptoms of fatigue, anxiety and depression were correlated with lower HRQoL. Moreover, parallel mediation analyses suggested anxiety and depression partially mediated the impact of pain and fatigue on HRQoL. This supports the practice of treatments for PH targeting these symptoms collectively. Indeed, multimodal interventions have been proposed for other long‐term health conditions [49] and there is some evidence supporting this approach in adults with PH. An observational study of 15 adults with PAH who received a 4‐week cardiorespiratory training programme in addition to cognitive‐behavioural sessions (4–10 per patient) reported significant improvements in measures of HRQoL, anxiety, depression and functional capacity, which reflected distance walked at 6‐min walk test, workload of cardiopulmonary exercise testing, peak VO_2_ and pulse O_2_ [50]. Such an approach to care is also consistent with recommendations by the European Society of Cardiology and European Respiratory Society for the diagnosis of treatment of PH, which recognises a holistic approach to PH is beneficial [2].

While our findings demonstrated a significant total effect between HRQoL and fatigue or pain self‐efficacy, suggesting targeting these specifically may result in positive outcomes, serial mediation analyses implied a possible causal link involving anxiety and depression. Fatigue was observed as having an influence on HRQoL through depression and anxiety, as opposed to anxiety and depression. Although this finding needs to be interpreted with caution given the explorative nature of the analyses, it is consistent with the literature suggesting there is a bidirectional relationship between fatigue and depression, which can be difficult to disentangle or differentiate between symptoms. Indeed, tiredness, low energy, cognitive difficulties, slowing down and sleeping difficulties could all be symptoms of depression or fatigue. Differentiating between these two conditions is important in practice, for example, depression may be overshadowed by fatigue.

One network analysis investigating subjective fatigue involving 322 individuals with multiple sclerosis found fatigue was most strongly related to depressive symptoms, which in turn was more linked to anxiety [51]. While different causal mechanisms have been proposed to help explain the high concurrence between depression and fatigue, psychologically, one way to conceptualise the link could be to view fatigue as a symptom of the pathophysiological mechanisms of PH, which may prevent or deter individuals from engaging in valued activities, thus triggering or exacerbating low mood and negative thoughts [52]. Such depressive experiences may reduce motivation or confidence to engage in activities, and overtime reduce conditioning. This means fatigue could be associated with secondary symptoms of PH [53]. Fatigue may also pose as a barrier to engaging in treatment or self‐management, further perpetuating difficulties [54]. This cycle is somewhat supported by participant's responses to the FSS in the current study, as 97% explained their motivation is lower when fatigued. Moreover, fatigue was reported to interfere with many aspects of daily life by the vast majority.

Treatments for fatigue in other chronic conditions suggest physical and psychological therapies or medication can be effective interventions. The appropriate approach however can depend on the patient's preference, ability, and stage of their disease [55]. The evidence‐base for the effectiveness of PH‐related treatments where both depression and fatigue have been measured is severely lacking. There are some physical activity‐based intervention studies that have targeted fatigue in PH as an outcome. Our finding that 69% of patients reported exercise brings on their fatigue, could help to inform the interpretation of such results. For example, while it is common to aim for a reduction in symptoms of fatigue at the end of treatment [53], no significant changes could also be a positive outcome [56]. It may indicate fatigue severity remained constant despite an increase in activity levels, which allowed the individual to engage in valued activities contributing to other factors associated with HRQoL.

In contrast, our findings hint at the idea that pain self‐efficacy has an indirect impact on HRQoL through anxiety and then depression. This is also consistent with our understanding of the impact of pain, which can be associated with symptoms of anxiety and panic. Bodily symptoms, such as pain, can be misattributed, sometimes termed catastrophically misinterpreted, to a life‐threatening cause, leading to feelings of anxiety and an exacerbation of bodily symptoms. The individual may look to break this cycle through avoidance or seeking behaviours that tend to be helpful in the short‐term, but strengthen the link [57]. It is possible that patients avoid activities associated with their pain, contributing to a depression and fatigue cycle discussed previously. In terms of participant's responses to the pain self‐efficacy questionnaire, the largest group felt confident that they could enjoy things, engage in work and cope in most situations despite the pain. However, individuals tended to feel less able to socialise, engage in hobbies or leisure activity, live a normal lifestyle, or increase their activity. Research is needed exploring the proposed relationships between variables measured here utilising longitudinal designs which will allow us to examine mediational effects over time and therefore propose more definitive conclusions. The fact we used a cross‐sectional design means we cannot argue cause and effect.

Due to the modest sample size, we were unable control for salient variables that may impact HRQoL, such as WHO functional class or type of PH. This would have also not been possible due to the fact 41% of the sample did not know their functional class. Upon further analyses, much of this group were from the UK. One working hypothesis for this is that it reflects the provision of healthcare in the UK where there is less emphasis on patients needing to know this information compared to their counterparts in countries where the information is asked routinely by health insurers. Another variable to control could have been symptom phenotypes in PH. Analyses involving 60 women with PAH proposed three clusters (mild, moderate and severe symptom cluster phenotype) based on dyspnoea, fatigue and sleep [58]. It is possible the relationships observed in the current study differ in their strength and/or order depending on severity or nature of PH symptoms. On a similar note, it is likely that other symptoms associated with PH could be linked with the mediators assessed here. For example, cycles have been described involving fatigue, anxiety and poor sleep – which is a common difficulty reported by this clinical group [59]. For example, while fatigue may be associated with tiredness and the need for sleep, anxiety may prevent the individual from sleeping or experiencing restful sleep. In turn, poor sleep is a characteristic of depression; patients with PH who reported moderate to severe sleep disturbance have reported greater levels of depression and anxiety compared to those with no sleep difficulties [59]. It is easy to conceptualise how this cycle would negatively impact HRQoL. Given the current study was a secondary analysis and data was collected primarily to address another research question, we do not have all information that may be relevant to the current study such participants' type of PH, their levels of activity and (dis)ability and cause of pain.

A final limitation is the use of a measure on the impact and confidence to cope with pain rather than a tool assessing pain symptomology itself. More of a limitation of the PSEQ than our study design, questions on the measure are also framed to assume the person is experiencing pain, for example, “I can enjoy things, despite the pain” when the responder may not be experiencing pain in that setting. That being said, in this scenario participants can endorse “completely confident” meaning pain is not a current problem. Moreover, no participant in the current sample scored the maximum value on the measure meaning all were impacted by pain in some way.

To conclude, in a community sample of individuals with PH, a considerable proportion experienced clinical levels of anxiety, depression and fatigue, and reported difficulties in their confidence in managing pain. This supports the evidence base demonstrating the co‐occurrence of these symptoms in this clinical group, as well as the possibility that some individuals experience barriers to accessing appropriate support and management. The negative impact of pain and fatigue on HRQoL needs to be considered in care plans for all patients – particularly as none of the demographics investigated here correlated with measures of functioning. Results indicate multimodal interventions may be more helpful for improving HRQoL in PH. More specifically, there is preliminary evidence that targeting fatigue or pain, in addition to depression and anxiety could have a positive impact on HRQoL in this patient group.

## Ethics Statement

The initial randomised controlled trial was approved by the Cardiff University Psychology Ethics Committee (EC.22.12.13.6673R2A). Additional permission to analyse the data for the purpose of the current study was obtained from the University Research Ethics Committee at The University of Sheffield (064631).

## Author Contributions

Gregg H. Rawlings developed the concept of the study and was involved in data analysis and writing the report for publication. Abbie Stark developed the concept of the study and was responsible for data collection and made substantial contributions to the final write up. Iain Armstrong was involved in data collection and write up. Vlad Costin was responsible for data analysis and writing the report for publication. Andrew R. Thompson developed the concept of the study, made substantial contributions to data analysis, and provided feedback on the final report. All authors approved the final version for publication.

## Funding

The authors have not declared a specific grant for this study from any funding agency in the public, commercial or not‐for‐profit sectors.

## Conflicts of Interest

Dr Rawlings has received payment from Janssen‐Cilag Ltd for a presentation on depression and PH. The authors have no conflicts of interest.

## Data Availability

Data can be made available on reasonable request.
